# Website Usage and Weight Loss in a Free Commercial Online Weight Loss Program: Retrospective Cohort Study

**DOI:** 10.2196/jmir.2195

**Published:** 2013-01-15

**Authors:** Kevin O Hwang, Jing Ning, Amber W Trickey, Christopher N Sciamanna

**Affiliations:** ^1^Department of Internal MedicineThe University of Texas Medical School at HoustonHouston, TXUnited States; ^2^The University of Texas at Houston - Memorial Hermann Center for Healthcare Quality and SafetyHouston, TXUnited States; ^3^Department of BiostatisticsThe University of Texas M.D. Anderson Cancer CenterHouston, TXUnited States; ^4^Department of MedicinePenn State College of MedicineHershey, PAUnited States

**Keywords:** Internet, Obesity, Overweight, Weight loss, Adherence, Attrition

## Abstract

**Background:**

Online weight loss programs are increasingly popular. However, little is known about outcomes and associations with website usage among members of free online weight loss programs.

**Objective:**

This retrospective cohort study examined the association between website usage and weight loss among members of a free commercial online weight loss program (SparkPeople).

**Methods:**

We conducted a retrospective analysis of a systematic random sample of members who joined the program during February 1 to April 30, 2008, and included follow-up data through May 10, 2010. The main outcome was net weight change based on self-reported weight. Measures of website usage included log-ins, self-monitoring entries (weight, food, exercise), and use of social support tools (discussion forums, friendships).

**Results:**

The main sample included 1258 members with at least 2 weight entries. They were 90.7% female, with mean (SD) age 33.6 (11.0) and mean (SD) BMI 31.6 (7.7). Members with at least one forum post lost an additional 1.55 kg (95% CI 0.55 kg to 2.55 kg) relative to those with no forum posts. Having at least 4 log-in days, weight entry days, or food entry days per 30 days was significantly associated with weight loss. In the multiple regression analysis, members with at least 4 weight entry days per 30 days reported 5.09 kg (95% CI 3.29 kg to 6.88 kg) more weight loss per 30 days than those with fewer weight entry days. After controlling for weight entry days, the other website usage variables were not associated with weight change.

**Conclusions:**

Weekly or more frequent self-monitoring of weight is associated with greater weight loss among members of this free online weight loss program.

## Introduction

More than one third of US adults are obese [1]. Online weight loss programs represent a convenient and scalable resource for the prevention and treatment of obesity. In order to maximize the efficacy of such programs, it is important to identify specific features that promote favorable weight loss outcomes [2].

A positive association between website usage (program engagement) and weight loss has been demonstrated among participants enrolled in randomized trials and other prospective investigations [3-11]. But because those studies involved research volunteers who met strict eligibility criteria, results may have limited applicability to the general public. Instead of enrolling in a formal research study, individuals seeking to lose weight are more likely to join a commercial online weight loss program. Naturalistic evaluations of commercial online weight loss programs are needed to understand the benefits and limitations of these increasingly popular resources. While recent reports have described outcomes among paid subscribers to commercial online weight loss programs based in Australia [12], the United Kingdom [13], and Sweden [14], comparatively little is known about website usage and weight loss among members of *free* online weight loss programs. This is an important gap in the literature because consumers and health care providers strongly prefer free weight loss programs [15,16].

Therefore, we evaluated a naturalistic cohort of members of SparkPeople, which is a free online weight loss program based in the United States. Prior studies described the accuracy of advice [17] and types of social support [18] shared among SparkPeople members, as well as the positive association between use of the program’s online forums and perceived social support [19,20]. The purpose of this retrospective cohort study was to describe the magnitude of weight loss and examine the association between website usage and weight loss.

## Methods

Membership in the SparkPeople online weight loss program is free and supported chiefly through advertising revenue. Most members are from the United States. The main features are educational content, self-monitoring tools (for weight, diet, and exercise), and social support venues (discussion forums, blogs, and “Friend” relationships similar to general online social networks). Members use the program largely in a self-directed and self-paced manner. They are free to use website features and make weight entries at any time. As of 2009, members could also access components of the website via mobile applications, although data on mobile access were not included in the present dataset.

### Study Sample

Approximately 521,000 members joined the program during February 1 – April 30, 2008. Systematic random sampling produced the initial cohort of 26,582 individuals with a baseline weight. This de-identified dataset included all available follow-up data for these members through May 10, 2010.

Members were then excluded from analysis if they reported extreme outlying values for baseline characteristics: age greater than 100 years, weight less than 100 lb (45.4 kg) or greater than 800 lb (362.9 kg) with no follow-up weight entries, or height greater than 10 ft (3.05 m). These extreme outlier values were considered unrealistic, data entry errors, and/or not relevant to the analysis. Exclusion of 469 members with outlier values left an interim cohort of 26,113 members. Although we did not implement a filter for very short heights, the filtering process eliminated members with extreme height values, as the height range was 49-78 inches in the final cohort (n=1258) described below.

Self-reported weights may be inaccurate due to data entry errors, such as omitting, adding, or transposing digits. All 58,574 weight entries were analyzed for potential data entry errors with a series of automated and manual procedures. There were three automated filters (F1, F2, F3) for members with three or more weight entries, and a fourth automated filter (F4) for members with two or more weight entries. The first filter (F1) entailed fitting a second order polynomial regression line on the weight entries and dates; the distance of each point from the expected line (residual) was calculated. Points were flagged for visual evaluation if the residual was more than three times the standard deviation of the residuals for the individual record, and the difference between the actual and expected weight was greater than 10 lb (4.5 kg). The second filter (F2) flagged members that had a change of more than 2% body weight per day (whether over a short or long period of time). The third filter (F3) flagged members who had an absolute change of 50 lb (22.7 kg) or more between any two weight entries. The fourth filter (F4) flagged members with a weight change of 100 lb (45.4 kg) or more over the complete recorded period.

The automated filters identified 301 potentially erroneous weight entries among 248 unique SparkPeople members. Two independent observers (KOH and AWT) manually reviewed these 301 weight entries in the context of the other weight entries for a given individual (the trend) to determine whether the entry was erroneous. The observers demonstrated high interrater reliability, with an overall 95% agreement and Cohen’s Kappa .90. When the observers disagreed about a weight entry, they discussed and reached a consensus determination. After the automated filters and manual review, 73 of 301 weight entries (24%) were deemed erroneous and excluded from analysis. This represents 0.12% of all weights available (73/58,574).

Because the main objective was to evaluate the relationship between website usage and weight loss, 20,518 members with only one weight entry were excluded. The subsequent interim cohort of 5595 members had at least two weight entries. Members with at least two weight entries were younger, with a mean (SD) age 35.3 (11.5) years vs. 37.2 (13.4) years, *P*<.001, heavier, with a mean (SD) BMI 31.6 (7.7) vs. 30.8 (20.2), *P*<.001, and more likely to be female (91.3% vs. 85.9%, *P*<.001) than those with only one weight entry.

**Figure 1:**
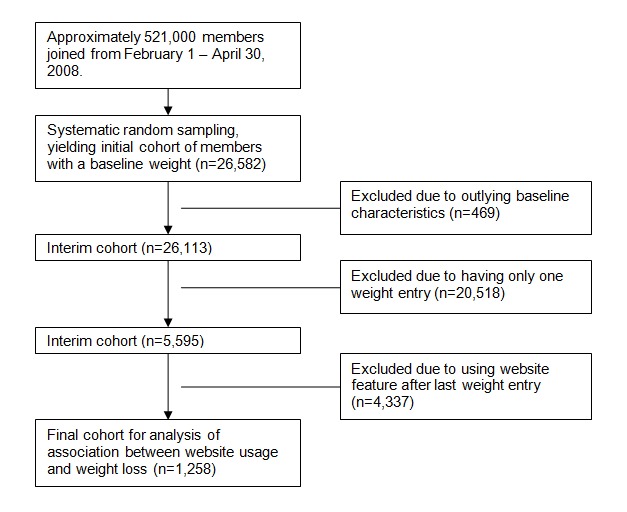
Flow diagram.

Each weight entry and log-in event was date-stamped. Because the data did not include the dates for use of other website features (eg, exercise diary), it was possible that a member used a website feature after his or her last weight entry, thereby complicating the interpretation of associations between website usage and weight change. However, it was possible to determine when a member stopped all website usage activity because each website activity generated a date-stamped log-in. Therefore, the final cohort of n=1258 was defined as those who had at least two weight entries, with the last weight entry on the same day as or after utilization of other website features. In other words, this final cohort consists of members who used the website features between their first and last weight entries. While the study design does not allow definitive analysis of causation, the final cohort at least meets the temporality criteria for causation. This final cohort was analyzed to examine the relationships between website usage and weight change. The flow diagram (Figure 1) depicts how the final cohort for analysis was defined.

### Definition of Website Usage Variables

Usage of website features included the following variables:

1. Log-in days: the number of days the member logged into the website at least once, regardless of whether s/he used any other website features while logged in.
2. Weight entry days: the number of days the member entered weight at least once.
3. Food entry days: the number of days the member made at least one entry in the food diary, regardless whether those entries were complete or incomplete.
4. Exercise entry days: the number of days the member made at least one entry in the exercise diary, regardless whether those entries were complete or incomplete.
5. Exercise minutes: the total number of minutes of exercise the member recorded in the exercise diary during the study period.
6. SparkPoints: the number of points earned by the member for miscellaneous website activities, such as reading articles, taking quizzes and polls, using food and exercise diaries, posting messages on forums, and making blog entries.
7. Forum posts: the number of messages the member posted on the discussion forums.
8. Friends: the number of other members designated as “SparkFriends” by the member (a social network feature).

### Analysis of Weight Entry Span and Weight Change

The weight entry span was defined as the number of days between first and last weight entry. Weight change, defined as the last recorded weight minus baseline weight, was stratified by weight entry span.

### General Analytic Approach to Website Usage and Weight Change

Potential confounders of the relationship between website usage and weight change included baseline BMI, age, gender, and weight entry span. Registration source (referred by friend, search engine ad, other) was examined as a potential confounder because it is possible that members who are introduced to the online program by a personal friend may differ in website usage and weight change than those who find the site only after an Internet search. Likewise, geographic location (zip code in midwest, northeast, south, or west region of the United States or other country) was examined as a potential confounder because of possible geographical variation in Internet use and dietary and physical activity factors related to body weight. These potential confounders were examined for association with weight change by using univariable regression analyses. Variables with a significant association (*P*<.05) with weight change were adjusted for in subsequent analyses.

Regression analysis was conducted with each website usage variable as predictor and net weight change as the outcome with adjustment for the identified confounders (univariable analyses). Multiple regression analysis with backward stepwise selection was conducted to identify the best subset of predictive covariates for net weight change. All covariates were included in the full model before model selection. Variables were included in the final model if *P*<.20.

### Analysis of Binary Website Usage Variables and Weight Change

The distribution of website usage was highly skewed, such that many members did not use a given feature before their last weight entry. Therefore, website usage variables were coded as binary variables for the initial analysis (*never* using a website feature vs. using it *at least once*). Binary coding of website usage has been used in a prior study of an online weight loss program [21]. Weight change was defined as the last recorded weight minus baseline weight. Because all members in the sample had two or more log-ins and weigh-ins, and most members had at least one SparkPoint, those variables were not included in this analysis.

### Analysis of Categorical Website Usage Variables and Weight Change

To further analyze associations between website usage and weight change, one option would be to assume a linear regression model, but the linearity assumption may be not be valid. For example, the change in weight associated with an increase from 3 to 4 forum posts may be different from the weight change associated with an increase from 150 to 151 forum posts. We considered categorizing the website usage variables into tertiles, but the data distributions were too skewed, so that observations in the top tertile may be equivalent to some observations in the middle tertile.

Weekly use of online weight loss program features has been associated with greater perceived social support [20] and weight loss [3,4,6,7,14]. Therefore, we categorized log-in days, weight entry days, food entry days, exercise entry days, and forum posts as greater than or less than 4 events per 30 days, corresponding to approximately once per week. The median value of SparkPoints was 20.8 per 30 days, so SparkPoints was categorized as above or below 20 per 30 days. We initially categorized exercise minutes as above or below 600 minutes per 30 days, corresponding approximately to 150 minutes per week, but there were not enough members who met this threshold to allow meaningful analysis. Therefore, we categorized exercise minutes as above or below 120 minutes per 30 days, corresponding to 4 exercise entries of 30 minutes length per 30 days. Zero use was also included as a category for all variables except log-in days and weight entry days because, by definition, all members in the analysis had at least one log-in day and weight entry day.

Weight change was defined as (last recorded weight minus baseline weight) divided by 30 day unit of weight entry span. For example, if a member’s last weight was 100 kg and baseline weight was 108 kg, and weight entry span was 60 days, then the weight change was -8 / 2 = -4 kg per 30 days.

Matlab version R2008b (MathWorks, Natick, MA) was used to identify potential outlying values for baseline characteristics and self-reported weights. The R statistical package (R Development Core Team, Version 2.14.1) was used for descriptive and regression analyses.

The study was approved by the Committee for the Protection of Human Subjects at the University of Texas Health Science Center at Houston.

## Results

Demographic characteristics of the final cohort are summarized in Table 1.

Most members did not use the website features frequently. Since values were skewed, they are summarized with medians and interquartile ranges in Table 2.

**Table 1:** Demographic characteristics of final cohort (n=1258).

Characteristics		mean (SD) or n (%)
**Age, years, mean (SD)**		33.6 (11.0)
**Gender, female, n (%)**		1141 (90.7)
**Baseline BMI, mean (SD)**		31.6 (7.7)
**Geographic location, n (%)**		
	US - Midwest	291 (23.1)
	US - Northeast	187 (14.9)
	US - South	400 (31.8)
	US - West	210 (16.7)
	Other country	170 (13.5)

**Table 2:** Website usage characteristics of final cohort (n=1258).

	Minimum	25^th^ percentile	Median	75^th^ percentile	Maximum
Log-in days	2	3	7	23	654
Log-in span (number of days between first and last log-in days)	1	54	306.5	624.8	826
Weight entry days	2	2	2	4	156
Weight entry span (number of days between first and last weight entry days)	1	54	306	623.8	826
Food entry days	0	0	2	9	270
Exercise entry days	0	0	1	4	258
Exercise minutes	0	0	40	256.5	39130
Friends	0	0	0	1	74
Forum posts	0	0	0	0	2758
SparkPoints	0	22.3	85	330	16480

**Table 3:** Total weight change stratified by weight entry span^a^ among final cohort (n=1258).

Weight entry span	n (%)	Mean (SD) total weight change
Up to 30 days	261 (20.7%)	-1.08kg (3.09)
31-60 days	69 (5.5%)	-2.00kg (2.68)
61-90 days	39 (3.1%)	-1.98kg (3.46)
91-183 days	131 (10.4%)	-2.32kg (6.50)
184-365 days	210 (16.7%)	-1.97kg (9.27)
366-548 days	178 (14.1%)	-0.99kg (8.04)
549-829 days	370 (29.4%)	-1.38kg (10.5)

^a^ Weight entry span is the number of days between first and last weight entry.

Table 3 shows the total weight change stratified by weight entry span.

Baseline BMI was the only potential confounder significantly associated with weight change. Each additional unit of BMI at baseline was associated with an additional reported weight loss of 0.28 kg (95% CI 0.22 to 0.33 kg, *P*<.001) during the weight entry span. Age, gender, referral source, geographic location, and weight entry span were not associated with weight change. Further analyses of predictors of weight change are adjusted for baseline BMI.

### Website Usage as Binary Variables

In the analysis with binary website usage variables, having at least one forum post was the only website variable significantly associated with greater weight loss. Members with at least one forum post reported an additional weight loss of 1.55 kg (95% CI 0.55 kg to 2.55 kg) relative to those with no forum posts (Table 4).

**Table 4:** Associations between website usage variables (binary) and weight change^a^ in the final cohort (n=1258).

	N (%)	Coefficient	95% CI	*P* value
At least one food entry day	893 (71.0)	-0.35 kg	-1.28 kg to 0.58 kg	.47
At least one exercise entry day	677 (53.8)	-0.67 kg	-1.52 kg to 0.18 kg	.12
At least one exercise minute	741 (58.9)	-0.61 kg	-1.47 kg to 0.25 kg	.16
At least one forum post	291 (23.1)	-1.55 kg	-2.55 kg to -0.55 kg	.002
At least one Friend	547 (43.5)	-0.80 kg	-1.66 kg to 0.06 kg	.07

^a^ Website usage variables were coded as binary (at least once versus never). Analyses were adjusted for baseline BMI. Because all members in the sample had two or more log-ins and weigh-ins, and most members had at least one SparkPoint, those variables were not included.

### Website Usage as Categorical Variables

In the analyses with categorical website usage variables, weekly log-ins, weight entries, and food entries (at least 4 in 30 days) were associated with greater reported weight loss than less frequent use (Table 5). More exercise entry days per 30 days was also positively associated with weight loss. SparkPoints was associated with relative weight gain compared to having zero SparkPoints (although only 74 of 1258 members had zero SparkPoints). On multiple regression analysis including baseline BMI and all website usage variables, weight entry days was the only variable significantly associated with weight change. Members with at least 4 weight entry days per 30 days (weekly) reported an additional 5.09 kg weight loss per 30 days (95% CI 3.29 kg to 6.88 kg) relative to those with fewer weight entry days.

**Table 5:** Univariable analyses of associations between website usage variables (categorized) per 30 days and reported weight change per 30 days^a^ in the final cohort (n=1258).

		Sample size	Coefficient	95% CI	*P* value
**Log-in days per 30 days**					
	0 to <4	842	reference		Overall: <.001
	≥4	416	-3.18 kg	-4.69 kg to -1.67 kg	<.001
**Weight entry days per 30 days**					
	0 to <4	1018	reference		Overall: <.001
	≥4	240	-5.09 kg	-6.89 kg to -3.29 kg	<.001
**Food entry days per 30 days**					
	0	365	reference		Overall:.002
	1 to <4	684	1.04 kg	-0.59 kg to 2.68 kg	.21
	≥4	209	-2.49 kg	-4.68 kg to -0.30 kg	.03
**Exercise entry days per 30 days**					
	0	581	reference		Overall: .021
	1 to <4	576	1.18 kg	-0.30 kg to 2.67 kg	.12
	≥4	101	-2.46 kg	-5.20 kg to 0.26 kg	.08
**Exercise minutes per 30 days**					
	0	517	reference		Overall: .054
	1 to <120	556	1.43kg	-0.12 kg to 2.98 kg	.07
	≥ 120	185	-0.89kg	-3.06 kg to 1.28 kg	.42
**SparkPoints per 30 days**					
	0	74	reference		Overall: .003
	1 to <20	546	5.35 kg	2.22 kg to 8.48 kg	<.001
	≥20	638	4.47 kg	1.37 kg to 7.58 kg	.005
**Forum posts per 30 days**					
	0	967	reference		Overall: .24
	1 to <4	233	0.99 kg	-0.86 kg to 2.84 kg	.30
	≥4	58	-2.09 kg	-5.52 kg to 1.33 kg	.23
**Friends**					
	0	711	reference		Overall: .55
	1	327	0.88kg	-0.81 kg to 2.57 kg	.31
	≥2	220	0.90 kg	-1.08 kg to 2.87 kg	.37

^a^ Analyses were adjusted for baseline BMI.

## Discussion

### Key Findings

Average weight loss, based on self-report, was modest in this free online weight loss program, but active users had better outcomes. Making weekly weight entries (at least 4 weight entry days per 30 days) was associated with an additional 5 kg weight loss. After controlling for weight entry days, the other website usage variables were not associated with weight loss.

### Comparison With Prior Studies

To our knowledge, this is the first analysis of a naturalistic cohort of members of a free online weight loss program available to the general public. Prior studies evaluated naturalistic cohorts of members who paid for monthly subscriptions to commercial online weight loss programs in Australia [12], the UK [13], and Sweden [14]. Because free programs may have greater potential to reach people in need of weight loss assistance [15,16], evaluating this free program is an important advancement in our understanding of weight loss resources. Another strength of this study was the use of multiple regression to identify variables that are independently associated with weight loss.

Our results are consistent with prior studies documenting a positive relationship between engagement in online programs and weight control. Most of these involved research volunteers and strict eligibility criteria [3-11], while others were naturalistic studies of commercial online programs [12-14]. Overall, favorable weight outcomes were associated with higher frequencies of log-ins [3-7,9,10,12,14], weight entries [6,10,11,14], self-monitoring entries for diet and/or exercise [6-8,10-13], and use of social support tools [6,8,12,13].

Although the current study discovered that several website usage variables were associated with weight loss, the multiple regression analysis indicates that weight entry days is the most important. It is possible that weight self-monitoring leads to frequent modification of diet and exercise behavior in response to weights. An alternative explanation is that members made weight entries only when they were losing (rather than gaining) weight. However, a post-hoc analysis found that 71% percent of members posted at least one weight reflecting a higher weight than a previous entry. In other words, they did not avoid documenting weight regain. The evidence in favor of weight self-monitoring improving weight control is strengthened by the consistency of the association in studies of online [6,10,11,14] and traditional [22-28] programs. When the analysis controlled for weight entry days, the other website usage variables were not related to weight change. It may be that the main benefit of these other website features is to maintain interest in the online program and maximize the opportunity for weight self-monitoring.

When website usage variables were coded as binary variables, making at least one forum post was associated with greater weight loss, although this analysis did not adjust for weight entry days. Because the content of the messages was not available for this study, we could not determine the nature of interactions on the forums. Furthermore, it was not possible to assess how often members read messages on a forum without posting (“lurking”). However, prior studies found that SparkPeople members receive high-quality advice [17] and social support [18] on the forums and that use of forums is associated with greater perceptions of social support [19,20]. Forum use may facilitate weight loss in part by boosting adherence to weight self-monitoring. Although the optimal source, frequency, and venue for social support has not been determined, the link between social support and weight loss has been reported in several studies of online [6,8,12,13] and traditional [29-34] weight loss programs.

### Limitations

The study had several limitations. First, attrition was high, which is a common problem plaguing commercial online weight loss programs [12-14]. The majority of members in the initial sample made only one weight entry, so that the main analytic sample represented a small portion of all who registered for the program. Compared to those with only one weight entry, members with multiple weight entries were younger, heavier, and more likely to be female—consistent with the profile of individuals who typically enroll in weight loss programs. Even within the analytic sample, 21% of members quit entering their weight after 30 days. Since it is very easy to join a *free* online program, it is possible that many people joined without a strong sense of commitment, thereby inflating the initial sample and the subsequent attrition rate. Sending personalized follow-up messages to inactive members may improve adherence to weight self-monitoring [7], although this approach would be difficult to implement without additional resources.

Another limitation of the study is that the available records did not include ethnicity, race, or clinical characteristics. Furthermore, members were predominantly female, as is typical of weight loss programs. Taken together, these limitations indicate that results cannot be extrapolated to the general population, regular Internet users, or those who initially who register for this online program. The study is also limited by reliance on self-reported weights. However, weight reported by members of another online weight loss program was highly correlated with and similar to objective weights [35]. The lack of date stamps on most website usage variables (except log-ins and weight entries) limited the depth of analysis on the intensity and patterns of website usage. Lastly, because this was an observational study, the causal link between usage of website features and weight loss cannot be definitively ascertained without experimental studies.

### Conclusions

The public health impact of an intervention is determined by efficacy and dissemination [36]. Because this online program is free, scalable, and widely disseminated, the potential public health impact is significant. The current study suggests that increasing the use of weight self-monitoring among current and future members may boost the impact of this program even more.
